# Multivalent electrostatic *pi*–cation interaction between synaptophysin and synapsin is responsible for the coacervation

**DOI:** 10.1186/s13041-021-00846-y

**Published:** 2021-09-08

**Authors:** Goeun Kim, Sang-Eun Lee, Seonyoung Jeong, Jeongkun Lee, Daehun Park, Sunghoe Chang

**Affiliations:** 1 Department of Physiology and Biomedical Sciences, Seoul National University College of Medicine, Seoul, 03080 South Korea; 2 Departments of Neuroscience and Cell Biology, Howard Hughes Medical Institute, Yale University School of Medicine, New Haven, CT 06510 USA; 3 UK Dementia Research Institute, University College London, Cruciform Building, Gower St, London, WC1E 6BT UK

**Keywords:** Synaptophysin, Synapsin, Liquid–liquid phase separation (LLPS), *Pi*–cation interactions, Synaptic vesicle cluster, Presynaptic nerve terminals

## Abstract

We recently showed that synaptophysin (Syph) and synapsin (Syn) can induce liquid–liquid phase separation (LLPS) to cluster small synaptic-like microvesicles in living cells which are highly reminiscent of SV cluster. However, as there is no physical interaction between them, the underlying mechanism for their coacervation remains unknown. Here, we showed that the coacervation between Syph and Syn is primarily governed by multivalent *pi*–cation electrostatic interactions among tyrosine residues of Syph C-terminal (Ct) and positively charged Syn. We found that Syph Ct is intrinsically disordered and it alone can form liquid droplets by interactions among themselves at high concentration in a crowding environment in vitro or when assisted by additional interactions by tagging with light-sensitive CRY2PHR or subunits of a multimeric protein in living cells. Syph Ct contains 10 repeated sequences, 9 of them start with tyrosine, and mutating 9 tyrosine to serine (9YS) completely abolished the phase separating property of Syph Ct, indicating tyrosine-mediated *pi*-interactions are critical. We further found that 9YS mutation failed to coacervate with Syn, and since 9YS retains Syph’s negative charge, the results indicate that *pi*–cation interactions rather than simple charge interactions are responsible for their coacervation. In addition to revealing the underlying mechanism of Syph and Syn coacervation, our results also raise the possibility that physiological regulation of *pi*–cation interactions between Syph and Syn during synaptic activity may contribute to the dynamics of synaptic vesicle clustering.

**Supplementary Information:**

The online version contains supplementary material available at 10.1186/s13041-021-00846-y.

## Introduction

Synaptic vesicles (SVs) form tightly packed clusters that are well distinguished from the surrounding cytoplasm. Recent studies have suggested that principles of liquid–liquid phase separation (LLPS) may underlie the organization of such clusters [1, 2]. LLPS is a process through which proteins, RNAs, and organelles can self-assemble into biomolecular condensates via multivalent, low-affinity interactions often involving intrinsically disordered regions (IDRs) of the participating proteins [3–5].

Synapsin (Syn), a major constituent of the matrix that connects SVs, was shown to have LLPS properties and to capture small lipid vesicles into its liquid phase in vitro [2]. We, however, recently found that unlike in vitro, Syn alone had a diffuse cytosolic distribution in living cells. We found that co-expression of Syn together with synaptophysin (Syph), an integral tetraspanin SV membrane protein, is required to induce the formation of biomolecular condensates in living cells. These condensates are indeed clusters of small synaptic-like microvesicles, which are highly reminiscent of SV clusters [1]. Since there is no direct interaction between Syn and Syph, and we hypothesized that their interaction was due to electrostatic interactions between negatively charged C-terminal region (Ct) of Syph (pI = 3.91, charge at pH 7.4 = − 4.1) and positively charged Ct of Syn (pI = 12.02, charge at pH 7.4 =  + 19.9), but the exact nature of the mechanism underlying their coacervation remains to be determined.

The Syph Ct is 90 amino acids long and contains evolutionarily conserved 10 repeated sequences, and 9 of which start with tyrosine (Y-G-P/Q-Q-G) [6]. Interactions among aromatic amino acids such as tyrosine and basic residues such as arginine are known to play a role in phase separation by providing *pi*–cation interactions [7–9]. The electron-rich *pi* system above and below the benzene ring is partially negative and this negatively charged region of the quadrupole interacts with positively charged amino acids [10]. Besides, *pi*–*pi* stacking interactions among the aromatic rings can also drive LLPS [8, 11]. Glycine, proline, and glutamine, collectively known as “disorder-promoting amino acids” [12–14], are also abundant in the Syph Ct. These suggest that Syph Ct may have a propensity for phase separation by mediating networks of interactions either with itself or with other proteins via *pi*-mediated interactions.

Here we found that the coacervation between Syph and Syn is primarily governed by multivalent *pi*–cation electrostatic interactions among tyrosine residues of Syph Ct and positively charged Syn. We showed that Syph Ct can undergo LLPS via *pi*–*pi* interactions between themselves but only when incubated at non-physiologically high concentration in vitro or when assisted by additional interactions in living cells. We further showed that mutating 9 tyrosine residues to serine (9YS) in the repeated sequences of Syph Ct completely abolished the phase separating property of Syph Ct. Accordingly, Syph Ct 9YS mutant failed to coacervate with Syn despite this mutant retaining the negative charge of Syph, indicating that electrostatic *pi*–cation interactions rather than simple negative–positive charge interactions mainly govern the coacervation between them. Together with our previous results, current findings further showed that a minimal reconstitution system in fibroblast can be a powerful model to gain mechanistic insight into the assembly of presynaptic structures. Our results further raise the possibility that modulation of *pi*–cation interactions between Syph and Syn by interactions with various presynaptic proteins during synaptic activity may control the dynamics of synaptic vesicle clustering.

## Materials and methods

### Plasmid DNA construction

The mouse synaptophysin-EGFP (Syph-EGFP) plasmid was kindly provided by Dr. Jane Sullivan (University of Washington, Seattle, WA). The synaptophysin C-terminal (Syph Ct, amino acids 219–308) was PCR-amplified and cloned into mCherry-N1. pCRY2PHR-mCherryN1 (Addgene plasmid # 26866) was a gift from Dr. Chandra Tucker. Syph Ct-mCh-CRY2PHR was made by amplifying CRY2PHR (amino acids 1–498) and cloning into Syph Ct-mCherry. pCMV-CIB1-mCerulean-MP was a gift from Dr. Won Do Heo (Addgene plasmid # 58366), and CIB1 was replaced with Syph Ct to construct Syph Ct-mCer-MP. To make Syph (Ct)_2_-mCr-MP, the Syph Ct sequence followed by a flexible linker sequence (amino acid sequence: GSAGSAAGSGEF) was inserted before the Syph Ct-mCer-MP sequence. To construct Syph Ct-linker-mCer-MP, the 90 amino acids-long linker from human NHE6 (amino acids 628–701) was PCR-amplified and inserted between Syph Ct and mCer of Syph Ct-mCer-MP sequence. Syph 9YS-HA was derived from Syph-HA by conducting multiple rounds of site-directed mutagenesis in 9 tyrosine residues in Syph Ct (Y245S, Y250S, Y257S, Y263S, Y269S, Y273S, Y284S, Y290S, and Y295S) with custom-made primers (Macrogen, Seoul, South Korea) using *i*-pfu (iNtRON Biotechnology, Seoul, South Korea). Syph Ct 9YS was PCR-amplified and replaced Syph Ct of Syph Ct-mCh-CRY2PHR to construct Syph Ct 9YS-mCh-CRY2PHR. Syph (Ct)_2_ 9YS-mCer-MP was derived from Syph (Ct)_2_-mCer-MP by 9YS mutations as described above. FUS-RBD (amino acids 212–500) was PCR-amplified from the full-length FUS (Korea Human Gene Bank, South Korea) and subcloned into mEGFP-N1. The mCherry-synapsin Ia (mCh-Syn) plasmid was provided by Dr. Roger Tsien, (University of California, San Diego). Syph Ct from Syph Ct-mCherry was subcloned into a pSNAPf vector (N9183S, NEB). Syph Ct-mCh, Syph Ct 9YS-mCh, Syph (Ct)_2_-mCer-MP, Syph (Ct)_2_ 9YS-mCer-MP, SNAP-Syph Ct, and FUS-RBD-mEGFP were subcloned in pET28a vector having N-terminal hexahistidine (6xHis) tag to purify proteins. The fidelity of all DNA constructs was validated by DNA sequencing.

### Antibodies

Primary anti-bodies; anti-HA (MMS-101R, Covance, Princeton, NJ), and anti-mCherry (ab167453, Abcam, Cambridge, MA, USA). Secondary anti-bodies; Goat anti-mouse IgG (H + L) Cross-adsorbed secondary antibody, Alexa Fluor 488 (A-11001, Invitrogen, Carlsbad, CA, USA), and Goat anti-rabbit IgG (H + L) cross-adsorbed secondary antibody, Alexa Fluor 568 (A-11011, Invitrogen).

### Cell culture and transfection

COS-7 cells were grown in Dulbecco modified eagle medium (DMEM, Welgene, Seoul, South Korea) with 10% FBS (Gibco, Carlsbad, MD, USA), and 1% penicillin and streptomycin (Corning, Corning, NY, USA) in a 37℃, 5% CO_2_ humid incubator. For transfection, PEI Max (Polyscience, Warrington, PA, USA) was mixed with plasmid DNAs in a 1:4 ratio (w/v) and the mixture was incubated for 20 min at room temperature (RT). The culture medium was replaced with serum-free DMEM and the mixture was added to the cells and incubated for 3 h at 37 °C in a CO_2_ incubator. After incubation, the medium was replaced with a fresh complete medium.

### Fluorescence imaging

All live-cell imaging except FRAP was performed using a 60X oil immersion objective lens (Plan Apo NA 1.4) on a Nikon spinning disk confocal microscope (CSU-X1, Nikon, Tokyo, Japan) equipped with a Neo sCMOS camera (Andor Technology, Belfast, Ireland). During imaging, cells were incubated in Tyrode’s solution (136 mM NaCl, 2.5 mM KCl, 2 mM CaCl_2_, 1.3 mM MgCl_2_, 10 mM HEPES, and 10 mM glucose, pH 7.3).

*Light-activated CRY2PHR clusters formation*: For CRY2PHR activation, 500 ms pulses of photoexcitation were delivered 5 times with a 488 nm laser using the photo-stimulation module in the Nikon imaging software (NIS-elements), and a 560 nm laser was used for mCherry imaging. The 488 nm laser setting in our spinning disk confocal microscope, corresponding to ~ 200 μW (measured with optical power meter 8230, ADCMT, Saitama, Japan), is sufficient to drive rapid phase separation of CRY2PHR-tagged Syph.

*1,6-Hexanediol treatment*: COS-7 cells transfected Syph (Ct)_2_-mCer-MP were imaged every 2 s using a 405 nm laser. After acquiring the first five images, 3% 1,6-Hexanediol (240,117, Sigma) was added to the cells for 1 min and washed.

*FRAP*: Photobleaching was performed using Nikon A1 confocal microscope (Nikon) with a 60X oil immersion lens (1.40 N.A.) and Nikon imaging software (NIS-elements). Time-lapse images were acquired every 1 s during 5 s, and a selected droplet was bleached with a 405 nm laser (100%) for 1 s. Fluorescence recovery was subsequently imaged every 1 s during the first 30 s and then every 2 s for 2.5 min. Fluorescence intensity in the bleached region was measured over time, normalized to the initial value, and plotted using Prism 8 (GraphPad Software, San Diego, CA, USA).

### Immunocytochemistry

Transfected COS-7 cells were washed several times using pre-warmed Tyrode’s solution and fixed in a 4% paraformaldehyde with 4% sucrose for 15 min at RT and washed with PBS. The cells were permeabilized with 0.25% Triton X-100 in PBS for 5 min at RT and blocked with 10% BSA for 30 min at 37 ℃. Then, cells were incubated with primary antibodies diluted (1:1500) in 3% BSA in PBS at 4 ℃ overnight. The cells were washed with PBS 3 times and incubated with Alexa Fluor-conjugated secondary antibodies (1:2000) in 3% BSA in PBS for 45 min at 37 ℃.

### Protein purification

All proteins were expressed in *Escherichia coli* BL21 (DE3). Cells were grown at 37 °C in 2xYT medium with kanamycin (50 μg/ml) to A_600_ 0.6–0.8, followed by induction with 0.5 mM isopropyl-β-d-thiogalactopyranoside (IPTG) for 4 h at 37 °C or overnight at 16 °C. The cell pellet was collected by centrifugation and resuspended in a lysis buffer (50 mM NaH_2_PO_4_ [pH 8.0], 300 mM NaCl, 10 mM imidazole, 1 mg/ml lysozyme, 0.1 mg/ml DNase I, protease inhibitor cocktail (including 104 μM AEBSF, 80 nM Aprotinin, 4 μM Bestatin, 1.4 μM E-64, 2 μM Leupeptin and 1.5 μM Pepstatin A) (Roche, Mannheim, Germany)) in an ice bath. Resuspended cells were sonicated and rocked for 1 h at 4 °C with 0.5% n-lauroylsarcosine sodium salt. In the case of 6xHis-Syph (Ct)_2_-mCer-MP, cells were lysed using B-PER (ThermoFisher, Waltham, MA, USA), and proteins were purified using inclusion body solubilization reagent (ThermoFisher). After centrifugation, the supernatant was incubated with Ni–NTA chelating agarose beads (Incospharm, Daejeon, South Korea) at 4 °C. Proteins were eluted with a buffer containing 50 mM NaH_2_PO_4_ [pH 8.0], 300 mM NaCl, 250 mM imidazole. All proteins were quantified by SDS-PAGE and stored at − 80 °C.

### In vitro droplet imaging

The target concentration of protein was reached by mixing the purified proteins diluted with the elution buffer with proper PEG-8000 solutions. The protein mixtures were incubated for 5 min on ice before being placed in the chamber. Protein samples were injected into custom chambers assembled by attaching washed 18 mm coverslips to glass slides with double-sided tape. SNAP-Syph Ct was diluted in the elution buffer with proper PEG-8000 and 2 mM DTT (D9163, Sigma). Then, SNAP-Cell 505-Star or SNAP-Cell TMR-Star (NEB) was added to reach 40 μM final SNAP ligand concentration. In vitro droplets imaging was performed at RT using a 60X oil immersion objective (Plan Apo NA 1.4) on a Nikon spinning disk confocal microscope with 488 nm and 561 nm lasers for mEGFP and mCherry-tagged protein, respectively. Phase separation was confirmed by visual inspection and analysis using ImageJ software (NIH). Particles with a size less than 0.4 µm^2^ and circularity less than 0.8 were excluded.

## Results

### Syph Ct contains repeated regions and forms liquid droplets alone when incubated at high concentration in vitro

We have recently reported that unlike purified Syn, which can assemble into liquid droplets by phase separation in vitro, Syn alone has a diffuse cytosolic distribution when expressed in fibroblasts. Only when Syn is expressed together with Syph, they formed liquid droplets which trap small microvesicles into clusters in living cells [1]. We further demonstrated the importance of electrostatic charge interactions between them since the increase in the ionic strength of the buffer dissociated Syph Ct from Syn. We, however, found no evidence of physical interaction between them, and thus the underlying mechanism for their coacervation remains to be determined.

**Fig. 1:**
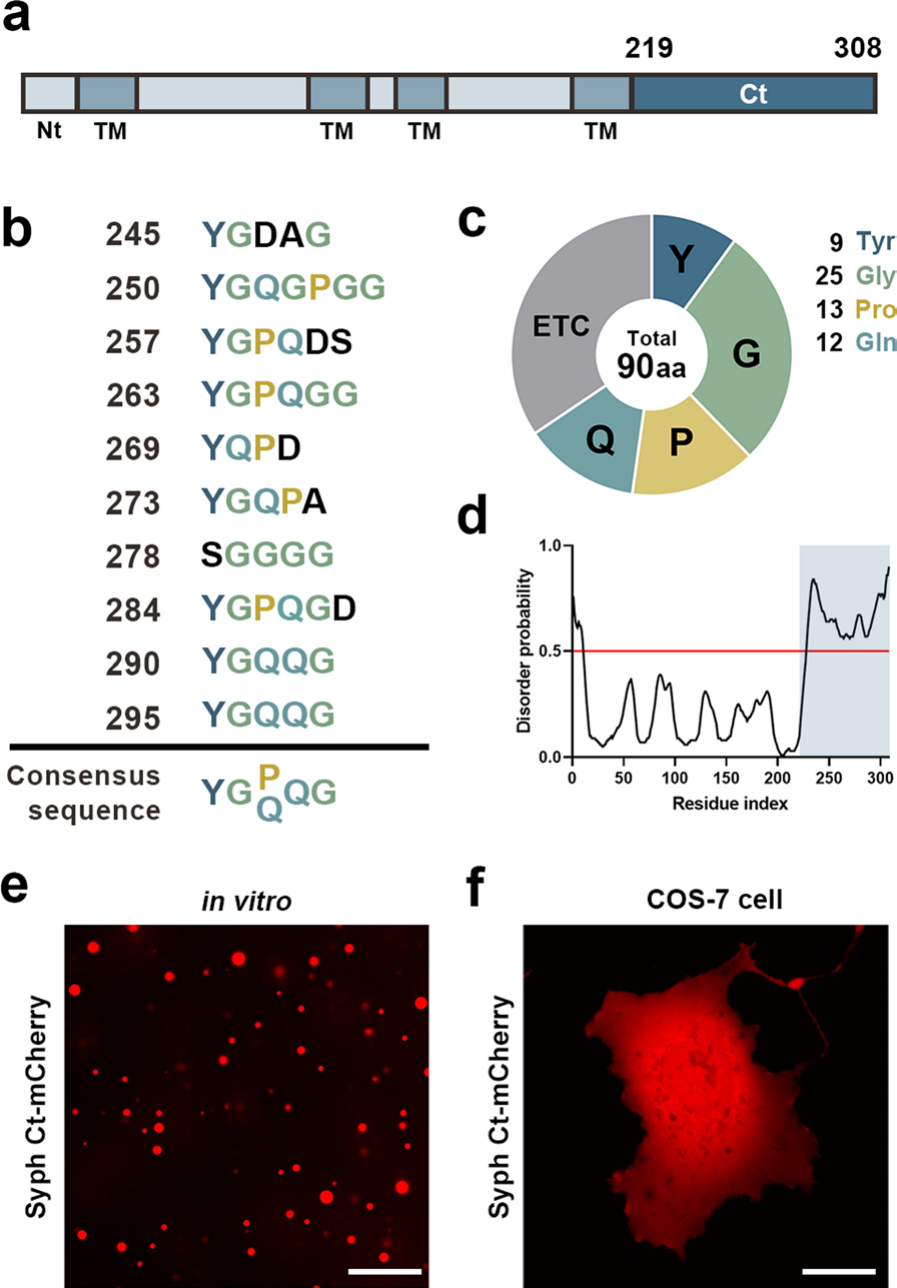
Syph Ct contains repeated regions and alone forms liquid droplets when incubated at high concentrations in vitro. **a** Domain structure of full-length mouse Syph. *Nt* N-terminus, *TM* transmembrane domain, *Ct* C-terminus (amino acids 219–308). **b** Repeated sequence in the cytoplasmic domain synaptophysin Ct. Syph Ct contains 10 repeated regions, 9 of which start with tyrosine. The repeated sequences are aligned to show the consensus sequence Y-G-P/Q-Q-G. **c** The pie graph shows the proportion of Tyr, Gly, Pro and Gln residue in the Syph Ct. **d** The prediction plot of intrinsically disordered regions in the full-length Syph using PrDOS. The shaded region is Syph Ct, which is likely to be an IDR. **e** Fluorescence images showing droplet formation of purified Syph Ct-mCh alone (50 μM) in vitro in the presence of 10% PEG-8000 at RT. **f** Representative fluorescence image of Syph Ct-mCh expressed in COS-7 cells. Scale bars, 20 μm

Syph Ct contains 10 repeated regions, 9 of which start with tyrosine [6] (Fig. 1a, b). Besides 9 tyrosine residues, glycine is the most frequently occurring amino acid (25/91, Fig. 1c). Glycine-rich regions are known as optimal spacers because they render conformational flexibility of the peptide bonds. Proline and glutamine are the next abundant amino acids (13 and 12/91, Fig. 1c). Proline acts as a structural disruptor of regular secondary structures and is known as the most disorder-promoting residue [14]. The glutamine residue, also known as a disorder-promoting residue, is required for the formation of labile cross-beta sheets [12]. Therefore, Syph Ct has a high propensity for phase separation, and is indeed predicted to be an IDR (Fig. 1d).

We previously showed that purified Syph Ct at 5 μM alone failed to form liquid droplets even in the presence of a crowding agent, PEG [1]. We have now found that further increasing the concentration of Syph Ct-mCherry to 50 μM (Fig. 1e) resulted in the formation of liquid droplets, indicating that Syph Ct can undergo LLPS in vitro although at non-physiologically high concentration. We also found that SNAP-tagged Syph Ct, formed liquid droplets in vitro in the presence of PEG (Additional file 1: Fig. S1).

When Syph Ct-mCherry was expressed in COS-7 cells, however, it did not form liquid droplets (Fig. 1f) even with extended-expression times. This does not rule out phase separating properties of Syph Ct, as other proteins, for example, synapsin undergoes phase separation only when incubated alone in a physiological buffer rather than in the cytoplasm of living cells.

### Syph Ct undergoes phase separation among themselves when assisted by additional interactions in living cells

Whether a system undergoes phase separation depends strongly on the local concentration of macromolecules [8]. We reasoned that the failure to form droplets in living cells could be due to the fact that the local concentration of Syph Ct by transient transfection did not reach the threshold concentration, the concentration above which the system starts to phase separate.

To determine whether Syph Ct could self-assemble in living cells if its local concentration increases, we employed the Opto-droplet system developed by Brangwynne group [15]. In this system, the protein of interest is fused to CRY2PHR, which undergoes clustering in response to blue light, but does not form droplets on its own. However, if the protein moiety fused to CRY2PHR has the propensity to self-assemble by phase separation, these clusters become nucleation sites for liquid droplet formation when light induces clustering of the fusion protein [15].

**Fig. 2:**
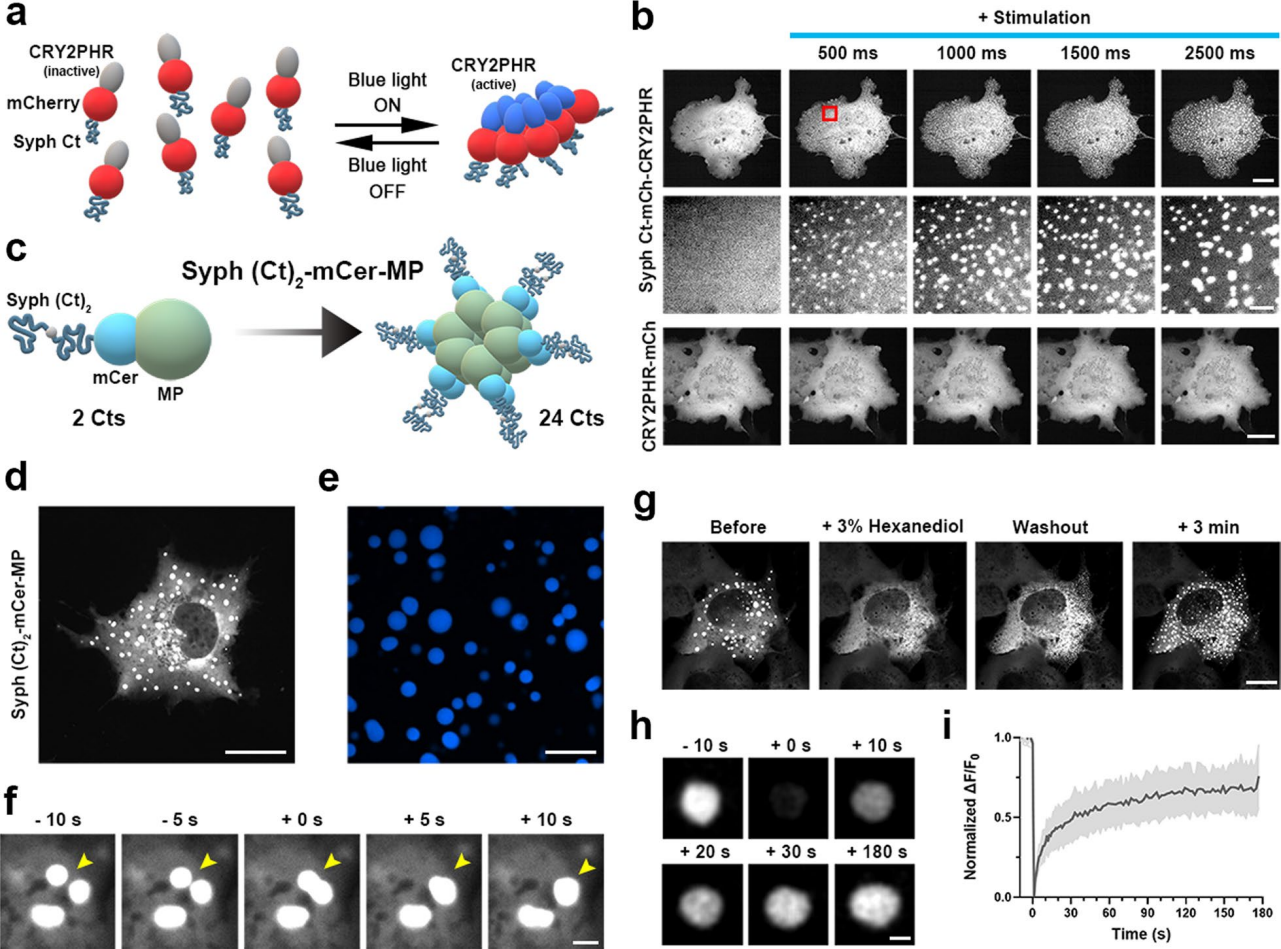
Syph Ct undergoes phase separation among themselves when assisted by additional interactions in living cells. **a** Schematic diagram of Syph Ct-mCh-CRY2PHR consisting of the N-terminal Syph Ct (blue-gray) fused to mCherry (red) and the CRY2PHR domain (gray indicating inactive state). Blue light activation of Syph Ct-mCh-CRY2PHR leads to rapid clustering (blue indicating active CRY2PHR). **b** Representative time-lapse fluorescence images of light-activated clustering of Syph Ct-mCh-CRY2PHR and CRY2PHR-mCh stimulated with a 488 nm laser for 2500 ms. Middle: Magnified images of the region enclosed by a red rectangle in the top panel. Scale bars; 20 μm (top and bottom), 2 μm (middle). **c** Schematic diagram of Syph (Ct)_2_-mCer-MP. Two Syph Cts were linked by a short linker (gray) and fused to mCerulean fluorescent protein and the multimeric protein (MP) of CaMKIIα (pale mint). 12 identical MP subunits are assembled into a circular oligomer, exposing 24 copies of Syph Cts. **d** Representative fluorescence image of droplets formed by Syph (Ct)_2_-mCer-MP expressed in living cells. **e** Representative fluorescence image of droplets formed by purified Syph (Ct)_2_-mCer-MP (5 μM) in vitro in the presence of 3% PEG-8000. Scale bars; **d** = 20 μm, **e** = 10 μm. **f** Time-lapse images showed fusion of two Syph (Ct)_2_-mCer-MP droplets in living cells. **g** Representative fluorescence images of Syph (Ct)_2_-mCer-MP droplets treated with 3% 1,6-Hexanediol (3% 1,6-HD). Droplets disperse reversibly upon 3% 1,6-HD. Scale bars; **f** = 2 μm, **g** = 20 μm. **h** Representative time-lapse images showing fluorescence recovery of Syph (Ct)_2_-mCer-MP droplet after photobleaching. **i** Plot of the average fluorescence intensities after photobleaching of multiple spots. N = 10 cells from 5 coverslips. Scale bars; 2 μm

We generated a chimeric protein consisting of Syph Ct, CRY2PHR, and mCherry (Syph Ct-mCh-CRY2PHR) and transfected it in COS-7 cells (Fig. 2a). Syph Ct-mCh-CRY2PHR showed diffuse cytosolic distribution in the dark but formed distinct droplets when briefly stimulated with blue light for 500 ms. Longer stimulation produced larger Syph Ct-mCh-CRY2PHR droplets (Fig. 2b), which remained after cessation of illumination. In contrast, CRY2PHR alone failed to form droplets even with extended stimulation for 2500 ms (Fig. 2b), indicating that CRY2PHR serves only as a nucleating mediator.

Syph is known to assemble into hexamers on the SV membrane and since ~ 30 copies of Syph are present in each SV, each SV contains 5–6 such hexamers [16–18]. To mimic such a high copy number of Syph in non-neuronal systems, we utilized the property of the C-terminal region of Ca^2+^/Calmodulin-dependent kinase IIα (CaMKIIα) that self-assembles into a circular oligomer of 12 identical subunits (called MP, a multimeric protein) [19]. Such oligomer has an outer diameter of about 30 nm, which well matches the average diameter of SVs (39.5 nm).

We first generated a Syph Ct-mCer-MP construct in which a Syph Ct was tagged to each MP subunit, and a circular oligomer contained 12 copies of Syph Ct. However, it failed to form liquid droplets when expressed in COS-7 cells (Additional file 1: Fig. S2).

We next generated a Syph (Ct)_2_-mCer-MP in which each mCer-MP was fused to two Syph Cts linked by a short linker and, thus a circular oligomer contained 24 copies of the Syph Ct (Fig. 2c). We found that it readily formed droplets in COS-7 cells (Fig. 2d). Simply increasing the length by inserting a linker (90 amino acids long, the same length as Syph Ct) between Syph Ct and MP failed to induce droplet formation (Additional file 1: Fig. S2). Purified Syph (Ct)_2_-mCer-MP also underwent phase separation in the presence (at 5 μM concentration, Fig. 2e) or absence (at 25 μM, Additional file 1: Fig. S3) of PEG at physiological salt concentration.

The liquid nature of Syph (Ct)_2_-mCer-MP droplets was confirmed by their property to coalesce into larger droplets (Fig. 2f) and also by 1,6-hexanediol (1,6-HD) treatment, aliphatic alcohol that disrupts weak hydrophobic interactions and dissolves LLPS droplets [20, 21]. We found that Syph (Ct)_2_-mCer-MP droplets dissolved within seconds after 3% 1,6-HD treatment and reformed rapidly upon 1,6-HD removal (Fig. 2g). Additionally, FRAP experiment showed that the fluorescence of Syph (Ct)_2_-mCer-MP droplets recovered rapidly after cessation of photobleaching (Fig. 2h, i), indicating the dynamic exchange of Syph (Ct)_2_-mCer-MP with those in the surrounding cytoplasm.

### Phase separation of Syph Ct alone is driven by tyrosine-tyrosine interactions

Syph Ct contains 9 tyrosine residues and multiple disorder-promoting residues. *Pi*–*pi* stacking interactions between the aromatic rings of tyrosine are known to drive phase separation [8, 11]. Indeed, we found that the PScore, a predictive score of the propensity of *pi*–*pi* interaction of a protein [11] of Syph Ct was 5.147 on average, which is higher than the confidence threshold (Additional file 1: Fig. S4). Therefore, we reasoned that tyrosine residues in Syph Ct are likely involved in self-interacting networks that lead Syph Ct to phase separation.

**Fig. 3:**
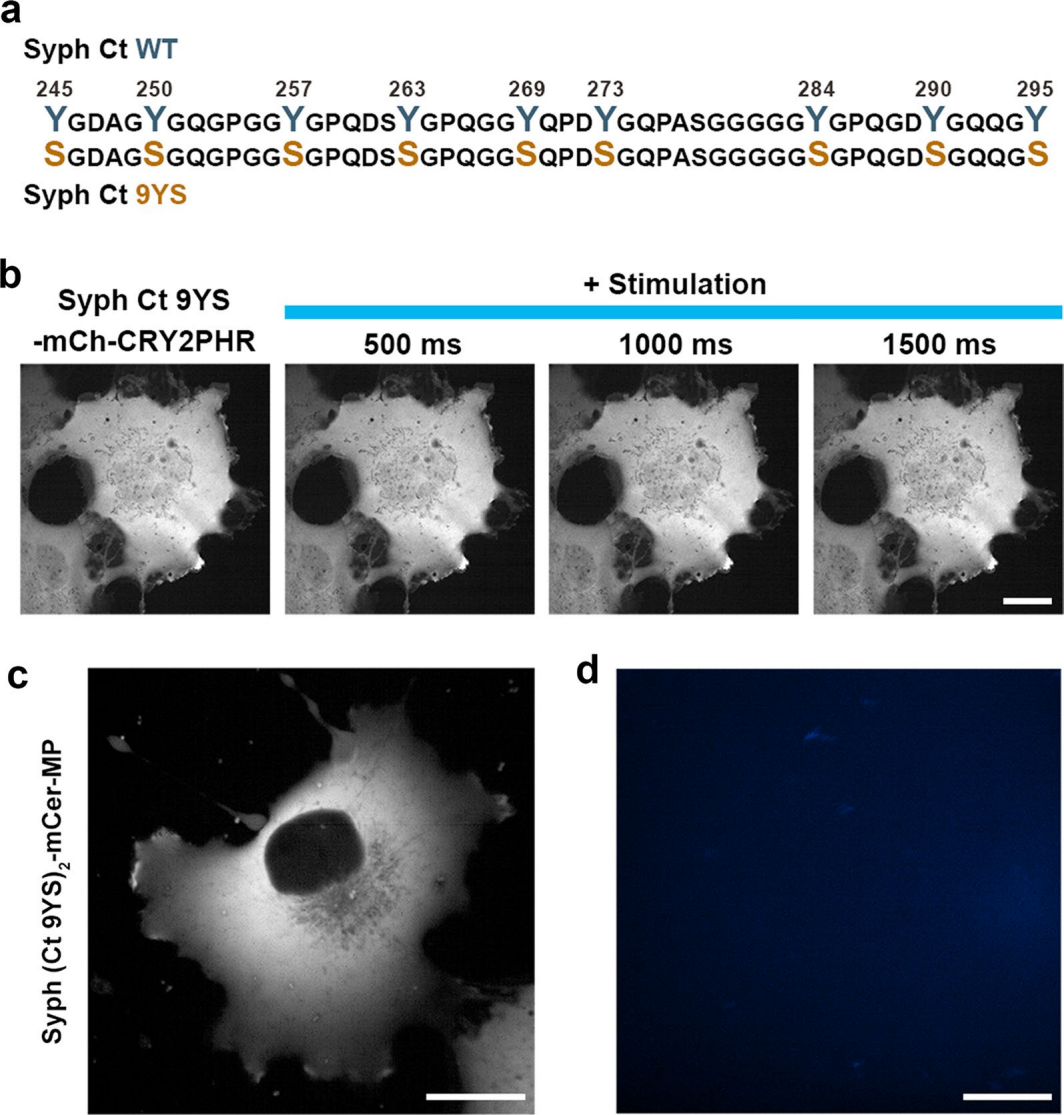
Phase separation of Syph Ct alone is driven by tyrosine-tyrosine interactions. **a** Schematic diagram of Syph Ct WT and 9YS mutant. Y245, Y250, Y257, Y263, Y269, Y273, Y284, Y290, and Y295 were mutated to serine (9YS). **b** Representative time-lapse fluorescence images of light-activation of Syph Ct 9YS-mCh-CRY2PHR in COS-7 cells. **c**, **d** Representative fluorescence image of Syph (Ct)_2_ 9YS-mCer-MP expressed in COS-7 cells (**c**) and purified Syph (Ct)_2_ 9YS-mCer-MP at 10 μM in vitro in the presence of 3% PEG-8000 (**d**). Scale bars, 20 μm

To test this possibility, we generated 9YS CRY2PHR and MP mutants in which all nine tyrosine residues (Y245, Y250, Y257, Y263, Y269, Y273, Y284, Y290, and Y295) were replaced with serine (Fig. 3a). We found that when expressed in COS-7 cells, Syph Ct 9YS-mCh-CRY2PHR did not form any droplets even with extended light stimulation (Fig. 3b). Likewise, Syph (Ct)_2_ 9YS-mCer-MP failed to form droplets in COS-7 cells (Fig. 3c) as well as in vitro (Fig. 3d). These findings are consistent with the possibility that phase separation of Syph Ct alone is mediated by *pi*-stacking interactions among tyrosine residues.

### Multivalent pi–cation electrostatic interactions tune the coacervating behavior between Syph and Syn

Purified Syph Ct could be forced to phase separate in vitro at a non-physiologically high concentration (50 μM) in the presence of PEG (Fig. 1e). However, Syph Ct alone was able to phase separate only when tagged with CRY2PHR or multimeric proteins in living cells (Fig. 2). These results suggest that phase separation of Syph Ct alone by *pi*–*pi* stacking may require a significant number of Syph Cts confined in very close proximity. In contrast, we hypothesized that *pi*–cation electrostatic attractions between tyrosine residues in Syph Ct and positively charged amino acids in other proteins could facilite coacervation between them.

**Fig. 4:**
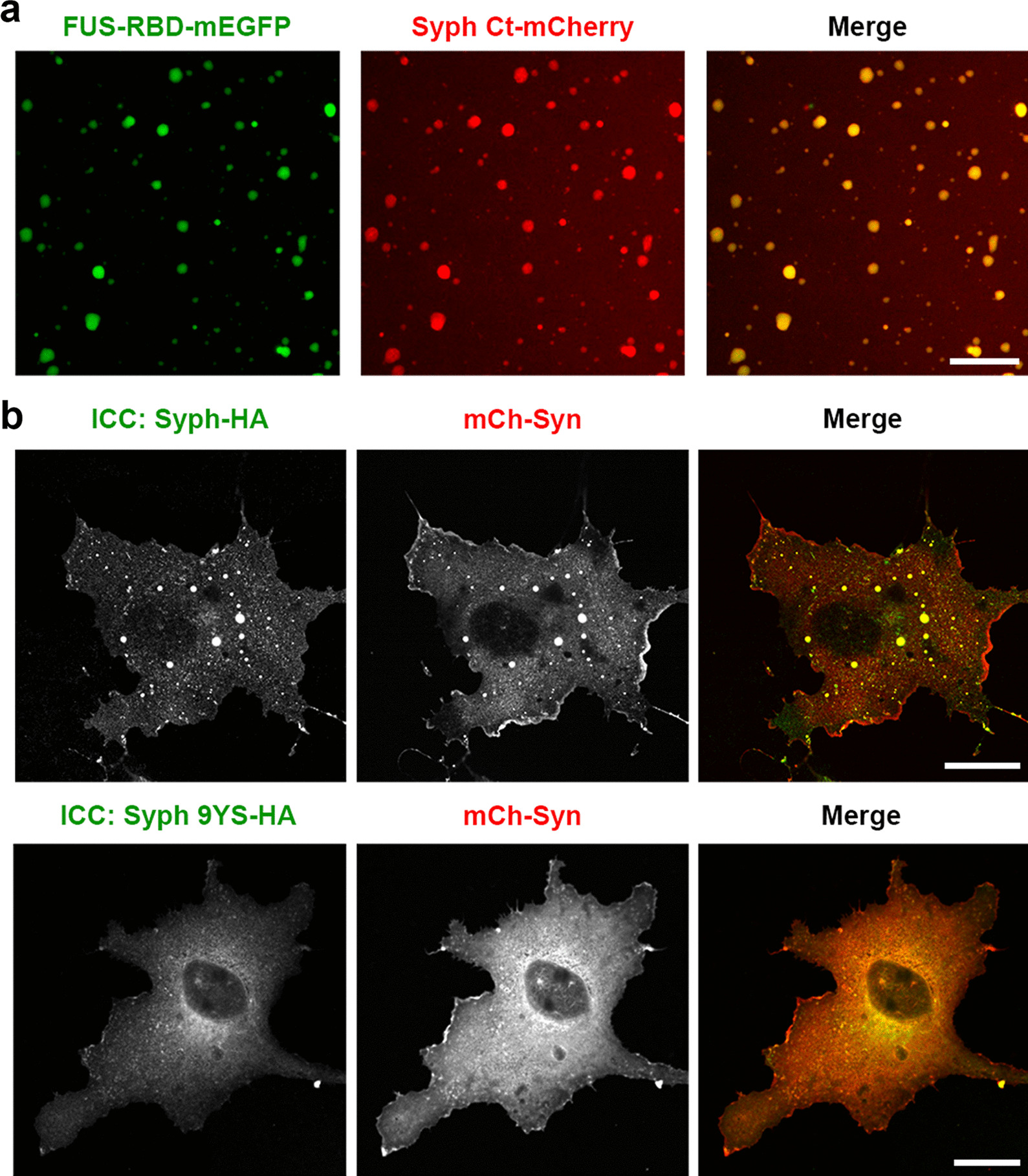
*pi*–cation electrostatic interactions govern the coacervating behavior between Syph and positively charged proteins. **a** Representative fluorescence images of co-condensates formed by purified Syph Ct-mCh and FUS-RBD-mEGFP in vitro. Syph Ct-mCh (5 μM) and FUS-RBD-mEGFP (2.5 μM) were mixed with 5% PEG-8000. **b** COS-7 cells were transfected with Syph-HA and mCh-Syn (top) or Syph 9YS-HA and mCh-Syn (bottom), and Syph was detected by immunostaining of HA (ICC). Unlike Syph-HA, no droplets were observed with Syph 9YS expression. Scale bars, 20 μm

To gain support to this hypothesis, we first co-expressed Syph Ct with Fused-in-sarcoma RNA binding domain (FUS-RBD). The major determinant of FUS-induced LLPS is known as the intermolecular *pi*–cation interactions between tyrosine residues in its prion-like domain (PLD) and multiple arginine residues in its RBD [8]. Thus, we used a FUS-RBD as a surrogate provider of positive arginine residues, expecting it to interact with tyrosine residues in Syph Ct via *pi*–cation interactions. We found that purified 5 μM Syph Ct and 2.5 μM FUS-RBD mixed at a physiological salt concentration in the presence of PEG readily formed co-condensates in vitro (Fig. 4a), suggesting that tyrosine residues in Syph Ct can mediate *pi*–cation interactions with positively charged residues, leading to phase separation at a much lower concentration than Syph Ct alone (50 μM, Fig. 1e). This is consistent with our previous results that 5 μM Syph Ct readily form co-condensate in vitro in the presence of PEG when mixed with Syn [1], indicating that the threshold concentration necessary to drive phase separation based solely on tyrosine residue-based interaction alone is at least an order of magnitude higher than that of tyrosine-basic residue-based interactions (50 μM vs. 5 μM).

Syn contains 85 positively charged amino acids and also has a polybasic C-terminal IDR that contains 31 positively charged residues, most of which are arginine (21/31) [1, 22]. To determine whether tyrosine residues in Syph Ct are critical for the coacervation between Syph and Syn, we co-expressed mCh-Syn with Syph-HA or Syph 9YS-HA in COS-7 cells. We found that while Syph-HA coacervated with mCh-Syn, which is consistent with our the previous results [1], Syph 9YS-HA failed to coaervate with mCh-Syn (Fig. 4b). Since we showed that tyrosine residues in Syph Ct can mediate *pi*–cation interactions with positively charged residues, and 9YS mutation retains the negative charge of Syph (− 8.3), these results are consistent with the possibility that multivalent electrostatic *pi*–cation interactions rather than simple negative–positive charge interactions mainly govern the coacervation between Syph and Syn in living cells.

## Discussion

We previously showed that expression of two presynaptic proteins, Syn and Syph in the cytoplasm of non-neuronal cells is sufficient to induce clusters of vesicles which are highly reminiscent of bona fide SV clusters in morphology and liquid properties [1]. Since there is no physical interaction between them, the underlying mechanism for their coacervation remains unknown. Here, we investigated the underlying mechanism leading to phase separation between Syph and Syn.

The characteristic 10 repeated sequences in the Syph Ct contain 9 tyrosine residues and we found that these tyrosine residues are critical for mediating either *pi*–*pi* interaction among themselves or *pi*–cation interactions with other positively charged proteins, leading to phase separation in vitro as well as in living cells. In addition to 9 tyrosine residues, glycine, proline, and glutamine are amino acids abundant in Syph Ct, all known as disorder-promoting residues [12, 14]. The phase behavior of associative polymers is governed by interactions through associative motifs called stickers, and the stickers are separated from one another by spacers, which are not the major determinants of the driving forces for phase separation [23, 24]. As in FUS [8], in Syph and Syn, stickers are tyrosine residues and positively charged residues, and abundant glycine and proline serve as effective spacers, to induce phase separation.

The *pi*–*pi* interactions are commonly associated with aromatic rings such as the side chains of tyrosine, phenylalanine, and tryptophan that provide *pi* orbitals of bonded sp^2^-hybridized atoms [11, 25]. Notably, low complexity IDRs implicated in phase separation of FUS, EWS, hnRNPA1, TIA-1, TDP-43, and the RNA Pol II C-terminal domain (CTD) [26–28] are very enriched in these residues and thus are highly likely to form *pi*–*pi* interactions. Syph Ct also contains 9 tyrosine residues, and the PScore, a predictive score of the propensity of *pi*–*pi* interaction of a protein [11], of Syph Ct was higher than the confidence threshold (Additional file 1: Fig. S4).

Indeed, we showed that the artificial clustering of multiple Syph Cts either by CRY2PHR-tagging or by MP tagging induced LLPS of Syph Ct alone in vitro and living cells (Fig. 2). This also is consistent with our previous result that Syph-EGFP readily formed liquid droplets when tagged with EGFP due to EGFP’s dimeric nature [1]. The importance of the *pi*–*pi* interactions between tyrosine residues in Syph Ct was further corroborated by our results that 9YS mutation completely abolished the phase-separating property of Syph (Fig. 3).

*pi*–cation interactions are noncovalent molecular interactions between the faces of electron-rich *pi*-systems and adjacent cations, and are recently known to be an important driving force for LLPS [7, 9, 10]. FUS has 27 tyrosine in the PLD, and *pi*–cation interactions between tyrosine residues in the PLD and arginine residues in the RBD mainly contribute to the phase separating behavior of FUS [8, 29]. Accordingly, an increase in the number of *pi*–cation interactions by arginine substitutions in FUS protein significantly promoted its ability to phase separate and lowered the threshold concentration of phase transition [8]. Our results using FUS-RBD as a surrogate provider of positive arginine residues proved that tyrosine residues in the Syph Ct can mediate *pi*–cation interactions with other positively charged proteins, leading them to coacervate (Fig. 4a). Syn also contains 85 positively charged amino acids and has a polybasic C-terminal IDR (pI = 12.02) that contains 31 positively charged residues, most of which are arginine [22]. Although we were not able to mutate all positively charged amino acids in Syn, which is practically impossible, since we showed that (1) tyrosine residues in Syph Ct can mediate *pi*–cation interactions with positively charged residues, (2) in contrast to wild-type Syph, co-expression of Syph 9YS with Syn completely abolished the formation of co-condensates (Fig. 4b), and (3) 9YS mutation retains the negative charge of Syph (− 8.3), our results are consistent with the possibility that multivalent electrostatic *pi*–cation interactions rather than simple negative–positive charge interactions mainly govern the coacervation between Syph and Syn in living cells.

Many SV proteins are predicted to contain IDRs. Synaptotagmin1 and VAMP2, are single transmembrane domain proteins that also have IDRs in the cytoplasmic region. Especially, VAMP2 is known to interact with Syph via its cytoplasmic region to form Syph-VAMP2 heterodimers [30, 31]. It is noteworthy that Syph KO studies did not find any obvious defects in synaptic physiology including SV clustering [32, 33]. These findings indicate that the function of Syph could be redundant with that of other SV proteins and thus the SV clusters may be dynamically regulated by different LLPS compositions formed by different presynaptic proteins depending on physiological needs.

Post-translation modifications and phosphorylation of various proteins are known to affect the LLPS behaviors [7, 34–36]. Rapid and reversible protein modification by phosphorylation can affect the intra- and intermolecular electrostatic interactions leading to LLPS by modulating the degrees of charge distribution of proteins. For example, LLPS propensity of FUS and TDP-43 decreases, but that of Tau LLPS increase depending on phosphorylation status [7, 35, 36]. Tyrosine phosphorylation also can disrupt *pi*-mediated interactions by increasing the negative charge of proteins. Indeed, previous study reported that tyrosine phosphorylation of Syph affects SV recycling during synaptic activity [37], thus whether phosphorylation of tyrosine in Syph Ct alters the degree of LLPS induction by shifting the electrostatic interaction with other proteins, and further the physiological significance of these alterations remain questions for future studies.

Although this reconstitution system in COS-7 cells does not fully reflect the phenomena at the presynaptic terminals and we cannot determine whether the interaction between Syph and Syn still has a significant effect on SV clustering in living neurons, the current study in addition to our previous study showed that a minimal reconstitution system is a powerful tool for investigating the mechanisms underlying the co-assembly between these two presynaptic proteins, otherwise would be complicated or hidden by interactions among various presynaptic proteins. Certainly, interactions among other synaptic proteins could either disturb or enhance the *pi*–cation interaction between Syph and Syn. Since *pi*–cation interaction is mostly based on the interaction between Tyr and positively charged amino acids, thus is venerable to physiologically relevant manipulations such as phosphorylation, pH variations, and protein interactions, we speculate that physiological manipulation of *pi*–cation interactions between Syph and Syn during synaptic activity may contribute to the dynamics of synaptic vesicle clustering. It is noteworthy that even in neuronal synapses, the correct stoichiometry for the interaction between Syph and other proteins including Syn is unknown. Besides, despite the results of the previous study [16], it is not yet known whether Syph forms actual hexamers in the SVs if so, how important it is for a physiological role. Thus, we are still far away from understanding what happens at real synapses and it certainly requires substantial further studies. In this regard, we believe that results from our previous and current studies using the minimal reconstitution system could provide meaningful clues to envision what might happen at real synapses, thus would trigger further studies in more physiological environments.

## Supplementary Information

**Additional file 1: Figure S1.** Purified SNAP-Syph Ct forms droplets in vitro as shown in Syph Ct-mCherry. **Fig. S2.** Single Syph Ct tagged to MP or increased length by inserting the linker failed to form droplets in COS-7 cells. **Fig. S3**. Purified Syph (Ct)_2_-mCer-MP forms droplets in vitro at a high concentration (25 μM) without crowding reagent. **Fig. S4**. The PScore plots of synaptophysin.

## Data Availability

All experimental protocols are described in the Materials and Methods section or the references therein, and resources are available upon request from the corresponding author SC.

## Abbreviations

Syph: Synaptophysin
Syn: Synapsin
LLPS: Liquid–liquid phase separation
Ct: C-terminal
SVs: Synaptic vesicles
IDR: Intrinsically disordered region
mCer: mCerulean
MP: Multimeric protein
NHE6: Na^+^(K^+^)/H^+^ exchanger 6
FUS-RBD: Fused-in-sarcoma RNA binding domain
6xHis: Hexahistidine
DMEM: Dulbecco modified eagle medium
FBS: Fetal bovine serum
RT: Room temperature
FRAP: Fluorescence recovery after photobleaching
IPTG: Isopropyl-β-d-thiogalactopyranoside
CaMKIIα: Ca^2^^+^/calmodulin-dependent kinase II
1,6-HD: 1,6-Hexanediol
PLD: Prion-like domain
CTD: C-terminal domain
