# Supplementary material for: Multivalent electrostatic pi–cation interaction between synaptophysin and synapsin is responsible for the coacervation
Source: Mol Brain. 2021 Sep 8;14:137. doi: 10.1186/s13041-021-00846-y (PMC8424992; doi:10.1186/s13041-021-00846-y)
Supplement: Supplementary file 1 — Additional file 1: Figure S1. Purified SNAP-Syph Ct forms droplets in vitro as shown in Syph Ct-mCherry. Fig. S2. Single Syph Ct tagged to MP or increased length by inserting the linker failed to form droplets in COS-7 cells. Fig. S3. Purified Syph (Ct)2-mCer-MP forms droplets in vitro at a high concentration (25 μM) without crowding reagent. Fig. S4. The PScore plots of synaptophysin. [file 13041_2021_846_MOESM1_ESM.pdf]

Additional Information for

Multivalent electrostatic *pi*-cation interaction between synaptophysin and synapsin is responsible for the coacervation

Goeun Kim[1], Sang-Eun Lee[1,#], Seonyoung Jeong[1], Jeongkun Lee[1], Daehun Park[2] and Sunghoe Chang[1,*]

[1]Department of Physiology and Biomedical Sciences, Seoul National University College of Medicine, Seoul 03080, South Korea, [2]Departments of Neuroscience and Cell Biology, Howard Hughes Medical Institute, Yale University School of Medicine, New Haven, Connecticut 06510, USA

*Address correspondence to: sunghoe@snu.ac.kr

This file includes:

Figure S1-S4

Figure S1. Purified SNAP-Syph Ct forms droplets *in vitro* as shown in Syph Ct-mCherry. Fluorescence images showing droplet formation of purified SNAP-Syph Ct (40 μM) *in vitro* in the presence of 10% PEG-8000 at RT. The SNAP-Syph Ct protein was stained SNAP-ligands, SNAP-Cell 505-Star or SNAP-Cell TMR-Star. Scale bars, 5 μm.

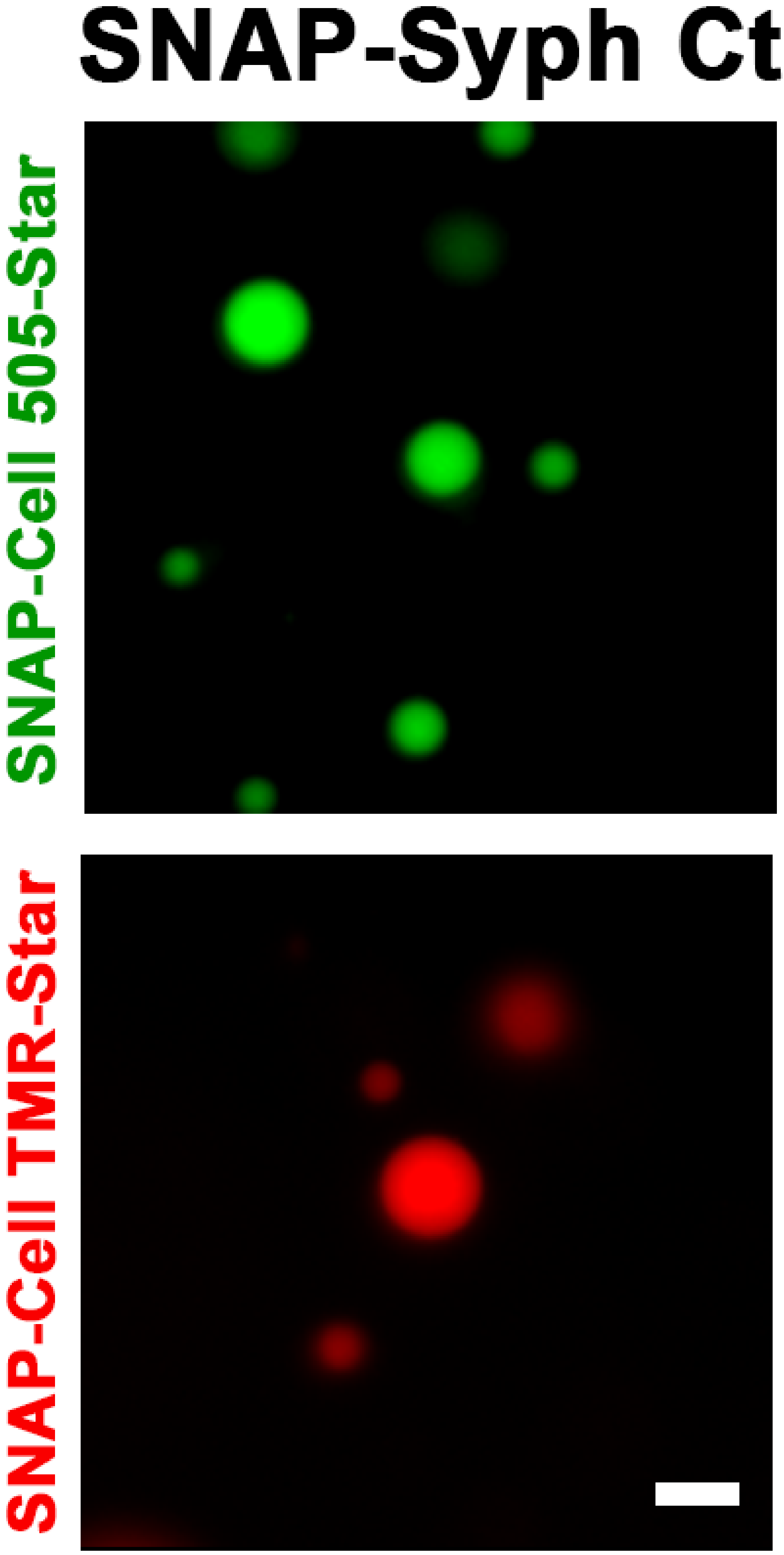
SNAP-Syph Ct
SNAP-Cell 505-Star
SNAP-Cell TMR-Star

Figure S2. Single Syph Ct tagged to MP or increased length by inserting the linker failed to form droplets in COS-7 cells.

a-c Schematic diagrams and fluorescence images of COS-7 cells expressed Syph Ct-mCer-MP (a), Syph Ct-linker-mCer-MP (b) and Syph $(Ct)_2$-mCer-MP (c). Syph Ct-mCer-MP failed to form droplets and increasing the length by inserting the linker (90 a.a. long, the same length as Syph Ct) between Syph Ct and MP also failed to induce droplet formation. Scale bars, 20 μm.

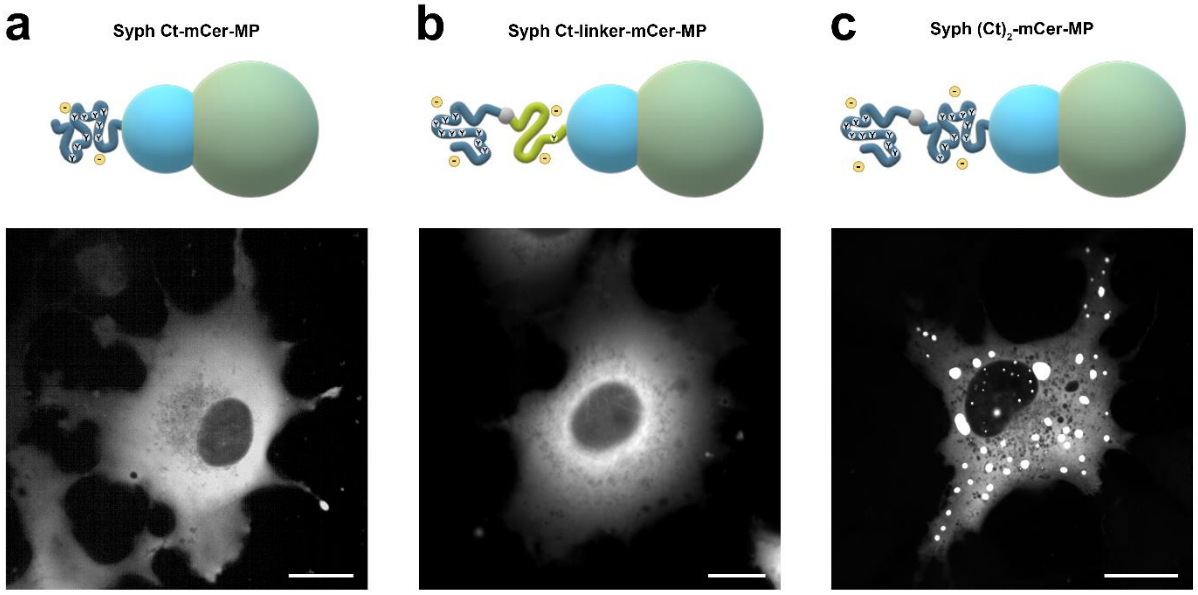
a
Syph Ct-mCer-MP
b
Syph Ct-linker-mCer-MP
c
Syph (Ct)2-mCer-MP

Figure S3. Purified Syph $(Ct)_2$-mCer-MP forms droplets *in vitro* at a high concentration (25 µM) without crowding reagent.

Representative fluorescence images of purified Syph $(Ct)_2$-mCer-MP at a high concentration (25 µM) *in vitro* in the absence of crowding reagent, PEG-8000. A series of magnified images of the regions enclosed by yellow rectangles (center → top right → bottom right)Scale bars, center: 20 µm, top right: 10 µm, bottom right: 2 µm.

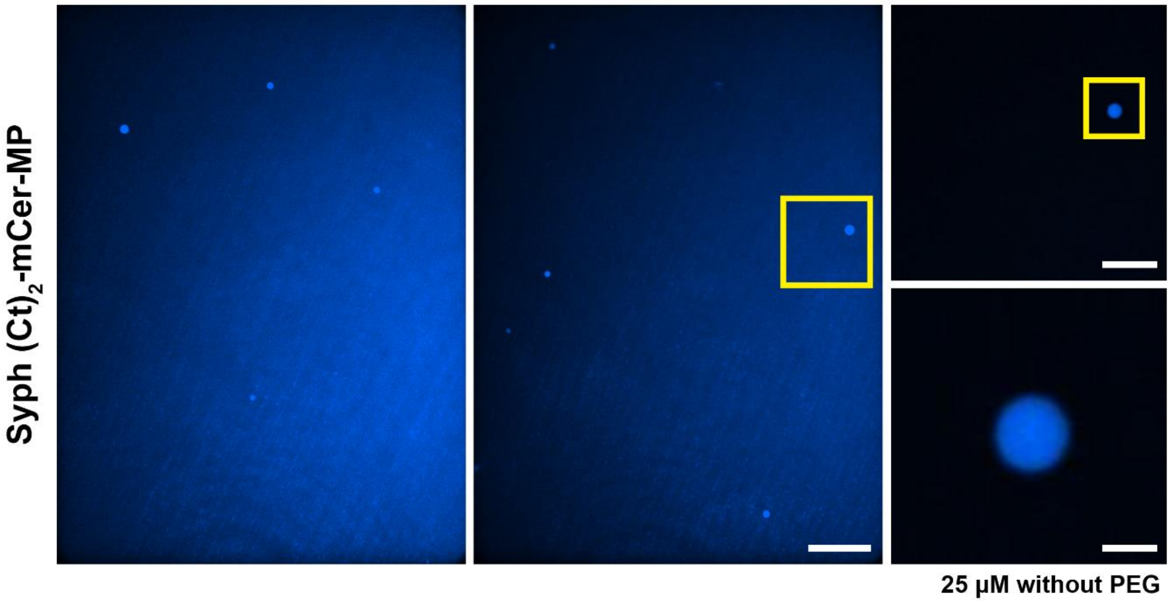
Syph (Ct)2-mCer-MP
25 µM without PEG

Figure S4. The PScore plots of synaptophysin

Heat map and a line graph of synaptophysin PScore, which is propensity score of *pi-pi* interaction tendency by prediction. X and Y axes are amino acids and PScore, and the average PScore of the repeat sequence in Syph Ct is 5.147.

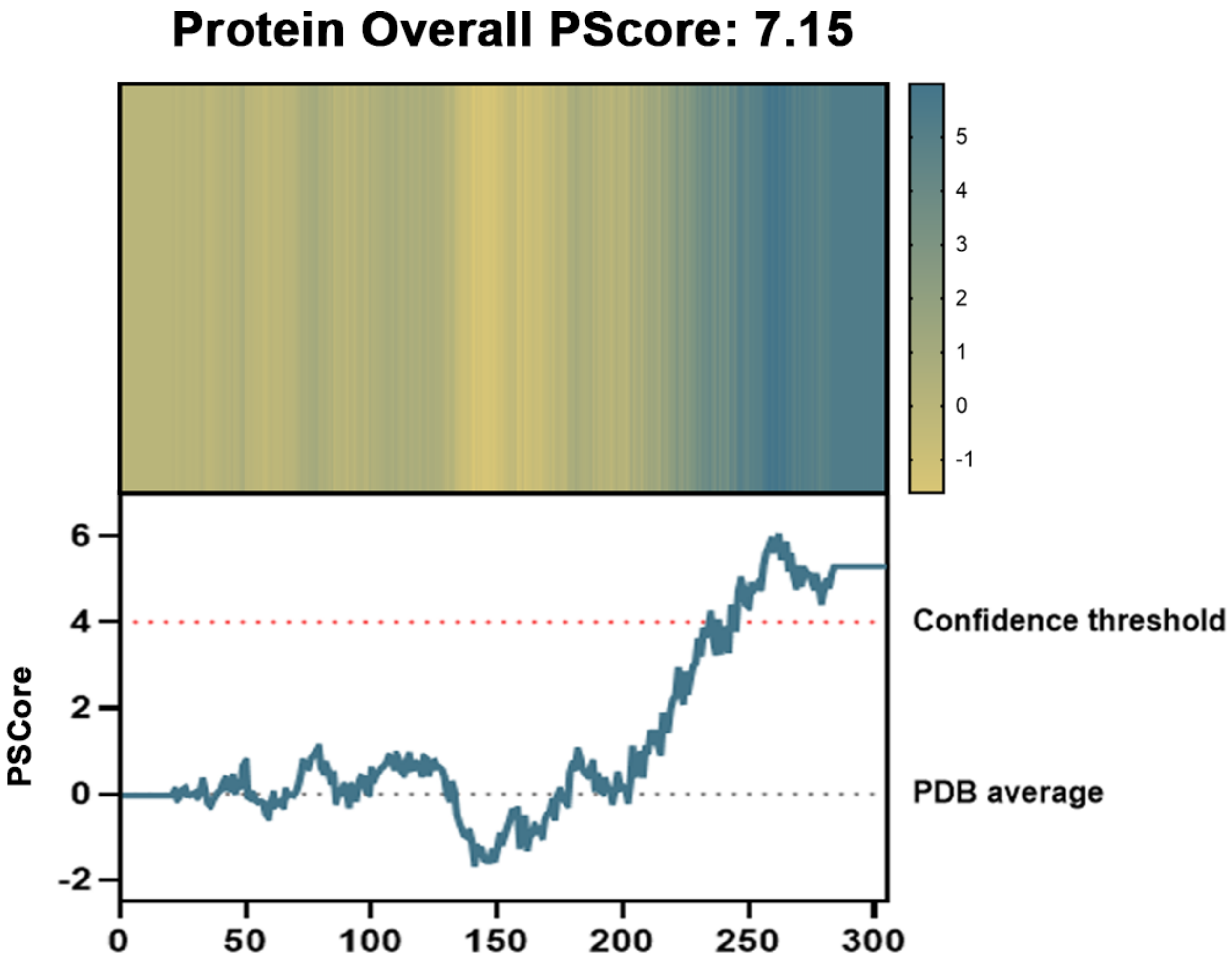
Protein Overall PScore: 7.15
5
4
3
2
1
0
-1
6
4
2
0
-2
PSCore
0
50
100
150
200
250
300
Confidence threshold
PDB average
